# The Conventions

**Published:** 1899-07

**Authors:** 


### Editorial.

### THE CONVENTIONS.

The period has arrived when the dental fraternity are looking forward to the annual gatherings, or, at least, such of the number who take an interest in these important meetings. It is unfortunately too true that only a small minority of the dentists of the country regard these with sufficient interest to take an active part in their deliberations. Why this is so, each individual must answer for himself, but that it is an error all recognize who appreciate their value individually and collectively. However defective these bodies may be, and it is recognized that they are far from perfect, they lead the thought of the dental profession, and without their aid there would not be a profession worthy the name. It must be accepted, therefore, as the duty of all to be present, and lend their aid by that presence, if for no other reason than to advance the standard of our calling, so that each year may bring it one step nearer to our ideals.

The present year promises to be one of unusual interest. The important question of legislation will be brought forward in, perhaps, all the conventions meeting at Niagara, and while it would be unreasonable to expect that all the difficult problems connected therewith will be settled, it is to be hoped that something will result determining the status of dentistry in its relation to law.

The National Dental Association, under the leadership of the energetic president, Dr. Burkhart, gives the promise of redeeming its early life, which came very near being shattered at Old Point Comfort and Omaha. The change made from the American and Southern, at the former place, failed to effect what all had anticipated by the union of the two organizations, but, probably, the mistakes then made will eventuate in something better than existed previously. It was the hope of the writer that upon the ruins of the two old organizations might be built up a body of men devoted to the higher work of dentistry, and an organization truly scientific in character. That this will come in time is assured, but it will only be accomplished by the gradual evolutionary process that a higher education can alone bring.

The preliminary announcement, made by the executive officers, exhibits, if nothing else, a wide interest and an earnest effort by a large body of men in the direction of better work. It evidences that the amount of material offered is altogether beyond the digestive powers of any convention, and this should be curtailed by the sections having the matter in charge. There are two positive evils connected with our annual gatherings,—a paucity of material and an excess. The first means a dull meeting, through lack of interest, and the latter an equally dull meeting by having more material than can possibly be presented. The time of a convention should be so subdivided that ample opportunity could be given for free expression of thought, for it is in this, more than from the papers, that the greatest good is to be derived. The life of every meeting is practically destroyed by the continuous reading of papers, and this is increased by the customary method of "reading by title."

While the making of constitutions is an infliction, and should not be entered into without due cause, it would seem that the laws under which the National Association is at present working should be, at least, modified. The constitution was adopted without due consideration at Old Point Comfort, and it needs entire revision and brought to the briefest possible limits. If the Association could see its way to effect this by referring it to a committee, to report next year, it would be of decided advantage to the Association and to its membership.

The National Association of Dental Faculties and the National Association of Dental Examiners will meet this year at the same place and time. The latter evidently has not found the holding of its annual meeting widely separated from the other active body a profitable venture. While these two organizations have little in common, the general idea of improving the profession is held by both, and it would, therefore, seem appropriate that the two meetings should be held near to each other. While consultations heretofore held through committees of both bodies have not resulted in harmonious action, it does not necessarily follow that this must always be the result. It is possible that a freer interchange of views might bring about better conditions and a more harmonious feeling.

The Association of Faculties has performed a great work in elevating dental education, and this is by no means ended; indeed, it has scarcely begun. The criticisms made against this body of earnest workers have had their origin in a class of mind that is never satisfied unless things are managed after their ideas. The cry has been repeated, over and over again, that this body reversed its decision upon preliminary training, and had taken a backward step, and had reached the limit of its power. Although this has been proven to be untrue, it serves the purpose of those in antagonism to dental colleges to repeat it with variations to suit the case.

The coming session of this Association will be of special importance, for many questions will be forced before it for consideration of vital interest to dental education. The dental colleges of the country are to-day between the upper and lower millstones of law and indifference of the profession, and some settlement must be made if harmony is to exist.

The National Association of Examiners is a body composed of delegates from State Boards. These are not all represented in its councils. Neither it nor the National Association of Dental Faculties have any legal standing. The latter works solely through the restraining power of its own laws upon its members. These have been found amply sufficient, and have accomplished much for the advancement of dental education. The Association of Dental Examiners works by another method,—that of binding the various State Boards to do its bidding, and through these to force the dental colleges to arrange their curricula according to the crude ideas of the National body. It is to be regretted that some of the colleges have seen fit to accept the decrees promulgated by this organization. Others have, very properly, refused to be thus bound, with the result that in some of the States the colleges and the boards are in a legal contest. So far the decisions have thrown the National Association of Examiners unceremoniously out of court. If this body is wise it will endeavor to work in harmony with the Faculties, and thus cease to accomplish the impossible. The colleges of repute in this country will not submit to be dictated to by an irresponsible body, and the earlier this fact enters the minds of the members of the National Association of Examiners the better it will be for all concerned.

The question of interstate recognition is being broadly considered, and it is one that must be met and answered. This is not the place to discuss it, and it will suffice here to say that the legislation of one State against another will not stand, but whether it will meet the test of legal decision or not is immaterial to the vital issue, which is, Is it equitable to deprive a man, having earned his diploma and passed the legal obstacles to practice, from making his living in any State of this Union? It is not only not equitable, but it is unprofessional.

There should be no sympathetic union with those who seek to embarrass the toilers in educational fields. They are the iconoclasts who aim to destroy the temple laboriously reared under the pretence of strengthening the foundations. They stand aloof, and in their ignorance demand the impossible, and thus throw these out of line with the educational structure and with the progress of the age.

